# Changes in eating behavior through lifestyle treatment in women with polycystic ovary syndrome (PCOS): a randomized controlled trial

**DOI:** 10.1186/s40337-022-00593-y

**Published:** 2022-05-17

**Authors:** Geranne Jiskoot, Alexandra Dietz de Loos, Reinier Timman, Annemerle Beerthuizen, Joop Laven, Jan Busschbach

**Affiliations:** 1 Division of Reproductive Endocrinology and Infertility, Department of Obstetrics and Gynecology, Erasmus MC, P.O. Box 2040, 3000 CA Rotterdam, The Netherlands; 2 Department of Psychiatry, Section Medical Psychology and Psychotherapy, Erasmus MC, P.O. Box 2040, 3000 CA Rotterdam, The Netherlands

## Abstract

**Background:**

Eating behaviors like emotional eating, external eating and restrained eating play an important role in weight gain and weight loss in the general population. Improvements in eating behavior are important for long-term weight. This has not yet been studied in women with Polycystic Ovary Syndrome (PCOS). The aim of this study is to examine if a three-component lifestyle intervention (LI) is effective for improving disordered eating behavior in women with PCOS.

**Methods:**

Women diagnosed with PCOS (N = 183), with a body mass index (BMI) > 25 kg/m^2^ and trying to achieve a pregnancy were either assigned to 1 year of 20 group sessions of cognitive behavioral therapy (CBT) combined with nutritional advice and exercise with or without additional feedback through Short Message Service (SMS) or Care As Usual (CAU), which includes the advice to lose weight using publicly available services.

**Results:**

The Eating Disorder Examination Questionnaire (EDEQ) scores worsened in CAU (47.5%) and improved in the LI (4.2%) at 12 months. The difference between the LI and CAU was significant (*P* = 0.007) and resulted in a medium to large effect size (Cohen’s d: − 0.72). No significant differences were observed in EDEQ scores between LI with SMS compared to LI without SMS (Cohen’s d: 0.28; *P* = 0.399). Also, weight loss did not mediate the changes in eating behavior. An overall completion rate of 67/183 (36.6%) was observed.

**Conclusions:**

A three-component CBT lifestyle program resulted in significant improvements in disordered eating behavior compared to CAU. Changes in disordered eating behavior are important for long-term weight loss and mental health.

*Trial registration*: NTR, NTR2450. Registered 2 August 2010, https://www.trialregister.nl/trial/2344

## Background

Polycystic Ovary Syndrome (PCOS) is a common endocrine disorder that affects 8–13% of women in their reproductive years [1–3]. The diagnosis of PCOS requires at least two out of three of the following criteria: (1) oligo-ovulation or anovulation (irregular or no menstrual cycle), (2) clinical hyperandrogenism (hirsutism) and/or biochemical signs of hyperandrogenism (elevated free androgen index or elevated testosterone levels), (3) polycystic ovarian morphology (by transvaginal ultrasound), and the exclusion of other etiologies that might cause hyperandrogenism [4]. Most women with PCOS experience one or more of the following symptoms: psychological (anxiety, depression, body image), reproductive (irregular menstrual cycles, hirsutism, acne and infertility) and metabolic (insulin resistance, metabolic syndrome, prediabetes and type 2 diabetes) [5, 6]. The prevalence of overweight and obesity is significantly higher in women diagnosed with PCOS compared to women without PCOS [6, 7]. Most women with PCOS have overweight or obesity throughout their entire lifespan [8, 9]. Weight loss can improve psychological, reproductive, and metabolic features of PCOS and is therefore the first line treatment [10].

Besides obesity, many women with PCOS experience depressive and anxiety complaints, have lower self-esteem and experience a more negative body-image compared to women without PCOS [11–13]. Other psychological aspects such as a lower quality of life and disordered eating have a major impact on women with PCOS [14].

Feeding and eating disorders, such as anorexia nervosa, bulimia nervosa and binge eating disorder (BED) are diagnosed according to the 5th version of the Diagnostic and Statistical Manual of Mental Disorders (DSM-5) [15]. Besides these official eating disorders, many individuals do not fulfill all the criteria of an eating disorder while having disordered eating patterns [16]. Disordered eating includes the full spectrum of eating-related problems like emotional eating, restrained eating and episodes of binge eating [17]. The Dutch Eating Behavior Questionnaire (DEBQ) was developed to measure three types of eating behavior: emotional eating, external eating and dietary restraint [18]. Emotional eating is defined as eating in response of stress or negative emotions [19] and is associated with overweight and weight gain [20]. Following a strict diet is considered a risk factor for emotional eating [21]. External eating is defined as overeating in response to the sight and smell of attractive food [22]. External eating is associated with a higher body mass index (BMI) and overweight. Emotional eating tends to co-occur with external eating [22]. Restrained eating refers to “chronic dieting” or intentional restriction of food intake to influence body weight, often interrupted with episodes of overeating (or eating more than wanted). After these periods of overeating or eating “forbidden” foods, restraint eaters tend to consume more in general [23, 24].

Most treatments for obesity are designed to increase dietary restraint. The problem of these restricted diets is that participants regain most of the lost body weight after stopping the diet. In fact, restricted diets work counterproductive with some patients even ending up weighing more than before the diet [25]. Restricted diets are also a significant contributor to binge eating [26]. Therefore, the treatment of people who have obesity should be tailored based on the type of eating behavior and focus on emotion regulation skills to achieve long term weight loss [22]. Cognitive behavioral therapy (CBT) is recognized as the first-line psychological treatment for obesity to develop healthy eating behavior [27].

and seems Also, CBT has proven to be effective for individuals with bulimia nervosa and binge eating disorder [28]. In women with PCOS, the odds for bulimia nervosa (OR 1.37), binge eating (OR 2.95) and any eating disorder (OR 1.96) are higher than in the general population [29]. Binge eating symptoms were more often present in women with PCOS compared to healthy controls [30]. Besides the increased odds for eating disorders, many women with PCOS have disordered eating behavior like emotional eating, dietary restraint and episodes of binge eating [31]. The odds for disordered eating were four times higher in women with PCOS compared to control [31]. Also, women with PCOS score higher on the Eating Disorder Examination Questionnaire (EDEQ) than women in the general population [31]. Especially the group of women with PCOS who also have obesity or high depression scores seems at risk for disordered eating [32]. Contrary to these results, Larsson and colleagues found no significant differences for restrained eating, uncontrolled eating, or emotional eating between women with PCOS and women without PCOS. However, women with PCOS did have higher scores on the Eating Attitudes Test compared with women without PCOS before and after adjustment for age and BMI [33]. This self-report questionnaire is used to assess the symptoms of eating disturbances and eating disorders. A higher score indicates more preoccupation with food consumption. This suggests that women with PCOS have greater weight and dieting concerns than women in the general population.

Weight loss by a three-component lifestyle intervention is recommended as first-line treatment for women with PCOS [34]. Compared to one or two-component lifestyle interventions, three-component lifestyle interventions have the biggest effect to establish a long-term weight loss in general [35]. These three-component lifestyle interventions should consist of: development of a healthy diet in combination with exercise and behavioral changes. Cognitive behavioral therapy (CBT) is used in obesity treatment as a technique for behavioral change. CBT is used for challenging and changing dysfunctional eating and body-related beliefs and schemas to develop and maintain a healthier eating pattern [36]. Important principles and techniques of the CBT component are self-monitoring, realistic and achievable goal setting, control of stressful stimuli and triggers, and promotion of alternative behaviors during emotional situations or negative mood states [37].

A lifestyle intervention (LI) was designed to examine the effectiveness of a 1-year three-component multidisciplinary program with or without Short Message Service (SMS) for women with PCOS and a BMI above 25 kg/m^2^. The mean weight loss was 2.32 kg in care as usual (CAU), 4.65 kg in lifestyle without SMS (LI without SMS) and 7.87 kg in lifestyle with SMS (LI with SMS). More weight loss was observed in LI compared to CAU (*P* < 0.001) and even more in LI with SMS compared to LI without SMS (*P* = 0.017) [38]. The current LI was designed to change behavior and achieve weight loss through a heathier lifestyle. Therefore, three hypotheses will be tested in this analysis of a secondary outcomes: (1) a three-component LI (with or without SMS) is more effective than CAU for improving disordered eating behavior, (2) LI with SMS is more effective than LI without SMS and (3) androgens, weight and depression mediate the effects of LI on disordered eating behavior.

## Material and methods

### Study design

We performed a longitudinal randomized controlled trial (RCT) measuring the effectiveness of a three-component multidisciplinary 1-year LI in women with PCOS and overweight or obesity. This study was approved by the Medical Research Ethics Committee of the Erasmus MC in Rotterdam; reference number MEC 2008-337 and registered at the Dutch Trial registration: reference number NTR2450. The current study on eating behavior represents an analysis of a secondary outcome. The results of the primary outcome (weight loss) and the design of the intervention have been described previously [38, 39].

### Participants

We conducted this RCT at the Division of Reproductive Endocrinology and Infertility, Department of Obstetrics and Gynecology of the Erasmus MC, Rotterdam, the Netherlands. Women were eligible if they were diagnosed with PCOS according to the Rotterdam 2003 consensus criteria, had a BMI above 25 kg/m^2^, between 18 and 38 years old and would like to become pregnant. Women with inadequate command of the Dutch language, severe mental illness, obesity with another somatic cause, ovarian tumors that lead to an androgen excess, adrenal diseases, had other malformations of their internal genitalia or who were pregnant, were not eligible for the study.

At baseline, and at 3-, 6-, 9- and during the follow-up at 12-months, all participants attended the outpatient clinic for standardized screening. All outcome measures were assessed during this screening. The screening included a family and reproductive history, anthropomorphometric and ultra-sonographic assessments. Participants also completed the Dutch Eating Behavior Questionnaire (DEBQ), EDEQ and Beck Depression Inventory-II (BDI-II) questionnaires at all these time points.

### Lifestyle intervention (LI)

The lifestyle treatment aimed at (1) changing cognitions through CBT; (2) developing healthy dietary habits; (3) encouraging and promoting physical daily activity, and; (4) activating social support. The intervention consisted of 20 group sessions of 2.5 h over the course of 12 months and was carried out by a multidisciplinary team. During the first three months, group sessions were scheduled every week. After three months, the interval changed to by weekly and after six months to once a month. During the last three months of the intervention, no meetings were planned. The first 1.5 h of every group session was supervised by a psychologist/CBT trainer and a dietician. The last hour of each session was supervised by two physical therapists. The Dutch Food Guide was used as a guideline for a healthy diet and daily amounts for the different food groups [40]. Participants were advised to make small changes in their daily life according to this guideline. No caloric restriction was advised. More information about which CBT techniques were used at each session and information about the daily amounts according to the Dutch Food Guide were described in the study protocol [39]. Drop-out is a well-known problem in lifestyle programs, therefore we used an outreach approach to motivate participants to come to the group meetings, unless the participant indicated to withdraw from the study. Participants were called or emailed several times when they were not present during a group-meeting to motivate them to come to the next meeting. Participants were allowed to be absence during 3 group meetings and needed to attend 17 out of the 20 meetings.

### Lifestyle intervention with additional short message service (LI with SMS)

After 3 months of LI, half of the participants in the LI received additional support by tailored SMS via their mobile phone. Participants sent weekly self-monitored information regarding their diet, physical activity and emotions by SMS to the psychologist. Participants received feedback on their messages to provide social support, encourage positive behavior and empower behavioral strategies. Besides, participants received two messages per week addressing eating behavior.

### Care as usual (CAU, control group)

The CAU group had 4 short, unstructured consultations with their treating physician during the standardized screenings at our outpatient clinic at 3, 6, 9 and 12 months. Participants in the CAU group were encouraged to lose weight through publicly available services (i.e. diets, visiting a dietician, going to the gym or participating in public programs such as Weight Watchers®). The physician also mentioned the risk of overweight for both mother and child, and the relation between overweight and fertility.

### Randomization

At baseline, participants were randomized at a 1:1:1 ratio using a computer-generated random numbers table by a research nurse. Participants who were assigned to either: (1) 20 CBT lifestyle group sessions including 9 months of electronic feedback through Short Message Service (SMS) via their mobile phone (LI with SMS) 2) 20 CBT lifestyle group sessions without SMS (LI without SMS) or 3) to the control group who received usual care (CAU). Written informed consent was obtained from all participants before the study. At baseline, 60 participants were randomized to CAU, 60 to LSLI with SMS and 63 participants to LSLI without SMS, resulting in a total of 183.

### Questionnaires

Eating behavior was measured with the Dutch Eating Behavior Questionnaire (DEBQ) and the Eating Disorder Examination Questionnaire (EDEQ). Depression was measured with the Beck Depression Inventory-II (BDI-II) at the start, 3, 6, 9 and 12 months:

The DEBQ [18] was used to assess eating in response to emotions with two dimensions called diffuse and clearly labeled emotions [13 items in total]. The diffuse emotion dimension (four items) and eating in response to clearly labeled emotions (9 items) can be summed. The two other subscales are: eating in response to the sight or smell of food (external eating, 10 items), and eating less than desired to lose or maintain body weight (dietary restraint, 10 items). This questionnaire consists of 33 items measuring 3 subscales. The subscale scores range between 1 and 5, with a higher score reflecting a higher degree of the relevant eating behavior. The EDEQ [41, 42] was used to measure specific eating disorders. The Dutch version consists of 36 items measuring 4 subscales: restraint, shape concerns, weight concerns, eating concerns, and a global score. The subscale scores range between 0 and 6. A higher score indicates more severe eating psychopathology. A global score or subscale score of 4 or higher is considered clinically significant. In women with PCOS, a mean EDEQ score of 2.38 has been reported compared to 1.29 in the general population [31].

The BDI-II is a validated and widely used questionnaire in depression trials assessing the severity of depressive symptoms over the previous 2 weeks, according to the DSM-5 criteria. The BDI-II is a 21-item self-report questionnaire with items rated on a 4-point scale (0–3) and are summed to give a total score (range 0–63). A higher score on the BDI-II denotes more severe depression. Scores of 0–13 indicate minimal depression, 14–19 (mild depression), 20–28 (moderate depression) and 29–63 (severe depression) [43].

### Statistical considerations

The power calculation was based on the primary outcome of the LI intervention: weight (kg). The method described by Aberson [25] was applied, with a power of 0.90, a 2-sided alpha of 0.025 (corrected for the interim analysis as described in the study protocol) and 5 repeated measures linearly decreasing. All variables were analyzed based on the intention-to-treat population, defined as all allocated participants. Multilevel or mixed regression modeling was applied for longitudinal outcomes. Mixed modeling can efficiently deal with missing data and unbalanced time-points [44, 45]. This means that, additionally, patients without complete follow-ups could be included in the analyses, without imputation. Study group, linear and logarithmic time and interactions were included as independent variables. The deviance statistic [46] using restricted maximum likelihood [47] was applied to determine the covariance structure thus taking into account the situation when e.g. the deviation at baseline is different from the deviations at follow-ups. In the case of a non-normal distribution, a bootstrap procedure with 10,000 samples was performed to obtain a more reliable outcome. The bootstrap mixed model analyses were performed utilizing IBM Corp (Released 2017. IBM SPSS Statistics for Windows, Version 25.0. Armonk, NY: IBM Corp). To test if weight, depression, androgens, insulin, the homeostatic model assessment for insulin resistance (HOMA-IR) and cortisol mediated the effect of LI on eating behavior, we used multilevel longitudinal mediation or indirect effect analyses. We selected these potential mediators based on a literature search, to limit the possibility of overfitting the mediation models. Paths α, β, τ and τ′ were estimated employing multilevel regression analyses. Firstly, we determined whether paths β were significant. When path β was not significant, mediation is unlikely. We adjusted the Sobel-Goodman test for the indirect effect of the independent variable on the dependent variable as reported by MacKinnon and Dwyer [48] following the recommendations by Krull and MacKinnon [49] for multilevel mediation analyses. The significance of the mediated effect is given by [50]:

$$Z_{mediation} = \frac{\alpha \beta }{{\sqrt {\beta^{2} SE_{\beta }^{2} + \alpha^{2} SE_{\beta }^{2} + SE_{\alpha }^{2} SE_{\beta }^{2} } }}$$

Cohen’s d effect sizes were calculated by dividing the differences between time-point and baseline estimations by the estimated baseline standard deviation. The guidelines of Cohen were used: effect sizes of 0.20 were considered as small, 0.50 as medium and 0.80 as large [51]. *P*-values < 0.05 were considered significant.

## Results

**Fig. 1:**
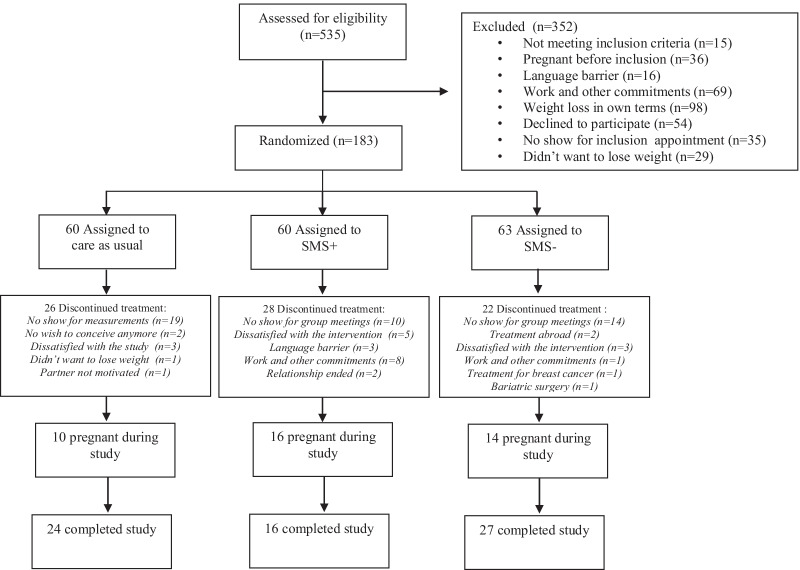
CONSORT flowchart

Between August 2nd 2010 and March 11th 2016, all 535 eligible women were asked to participate and 209 provided written informed consent, of whom 26 were included in a pilot study. At baseline, 60 participants were randomized to CAU, 60 to LI with SMS and 63 participants to LI without SMS, resulting in a total of 183. Of these 183 participants, 24 completed CAU, 16 completed LI with SMS and 27 completed LI without SMS. An overall completion rate of 67/183 (36.6%) was observed, Fig. 1. At baseline, 179 participants filled in eating behavior questionnaires. In total, 394 measurements were available for these analyses. The baseline characteristics of the participants are shown in Table 1.

**Table 1:** Baseline characteristics by trial group

	CAU (N = 60) Median [IQR]	LI without SMS (N = 61) Median [IQR]	LI with SMS (N = 58) Median [IQR]
EDEQ total	1.7 [0.13–2.7]	1.8 [0.0–3.1]	2.1 [0.8–2.8]
Restrained eating	1.4 [0.0–2.6]	1.2 [0.0–2.6]	1.3 [0.2–2.9]
Shape concern	2.4 [0.0–3.4]	2.6 [0.0–4.3]	2.8 [0.7–3.7]
Weight concern	2.1 [0.1–3.6]	2.4 [0.0–3.8]	2.7 [0.8–3.8]
Eating concern	0.4 [0.0–1.4]	0.2 [0.0–1.4]	0.8 [0.0–1.8]
DEBQ			
Emotional eating	2.4 [1.9–2.9]	2.9 [1.9–3.4]	2.7 [2.0–3.5]
Clearly defined emotions	2.3 [1.7–2.8]	2.7 [1.8–2.7]	2.7 [1.9–3.6]
Diffuse emotions	2.9 [2.0–3.3]	3.3 [2.0–3.9]	3.3 [2.3–4.0]
Restrained eating	3.2 [2.8–3.6]	3.0 [2.7–3.4]	3.1 [2.7–3.6]
External eating	2.7 [2.2–3.0]	2.9 [2.5–3.3]	2.9 [2.4–3.1]
Age (year)	28.0 [26.0–32.0]	30.0 [27.0–33.0]	28.0 [26.0–32.0]
Attempting to conceive (months)	24.0 [13.0–61.0]	24.0 [14.0–48.0]	20.0 [8.0–31.0]
Weight (kg)	84.0 [79.0–97.3]	89.0 [80.0–104.0]	94.5 [85.0–106.3]
BMI (kg/m^2^)	30.6 [29.3–34.4]	33.5 [30.5–36.0]	33.6 [31.0–36.8]
Weight loss (5%)	21.8 [8.5–45.5]	52.8 [23.2–80.5]	85.7 [51.3–97.2]
Weight loss (10%)	6.8 [1.7–23.5]	12.2 [3.2–36.7]	45.9 [15.4–79.8]
Modified Ferriman–Gallwey score	3 [1–6]	4 [2–9]	3 [1–9]

	N (%)	N (%)	N (%)
Binge eating episodes	25 (41.7)	36 (57.1)	25 (41.7)
Menstrual cycle			
*Oligomenorrhea*	51 (85.0)	52 (85.2)	37 (63.8)
*Amenorrhea*	6 (10.0)	7 (11.5)	17 (29.3)
*Regular*	3 (5.0)	2 (3.3)	2 (3.4)
Caucasian	19 (32.2)	20 (32.8)	26 (44.8)
Education			
*Low*	7 (11.7)	4 (6.6)	5 (8.6)
*Intermediate*	17 (28.3)	23 (37.7)	23 (39.7)
*High*	6 (10.0)	16 (26.2)	11 (19.0)

*IQR* interquartile range, *CAU* care as usual, *LI without SMS* lifestyle without short message service, *LI with SMS* lifestyle with short message service, *EDEQ* Eating disorder examination questionnaire, *DEBQ* dutch eating behavior questionnaire

### Changes in EDEQ scores

EDEQ global scores worsened in CAU by 47.5% and improved during LI by 4.2%. The difference between the CAU and LI group was significant (Cohen’s d: − 0.72; *P* = 0.007), Table 2. No significant differences were observed in EDEQ global scores between LI with SMS compared to LI without SMS (Cohen’s d: 0.28; *P* = 0.399). During the study period, no significant difference was observed for EDEQ restraint [A] (*P* = 0.254) and eating concern [D] (*P* = 0.116) between CAU compared to LI. Furthermore, the shape concern [B] (*P* = 0.016) and weight concern [C] subscale (*P* = 0.007) changed significantly between CAU compared to LI, Table 2. If we compared the difference between LI without SMS and LI with SMS, no differences were found for the EDEQ global score (*P* = 0.399), shape concern (*P* = 0.992) weight concern (*P* = 0.790) and eating concern (*P* = 0.954) between these two groups. Only for the restraint subscale, we found a significant difference between LI without SMS and LI with SMS (*P* = 0.015). During LI without SMS restrained eating scores remained stable while restrained eating scores worsened in LI with SMS, Table 2.

**Table 2:** EDEQ total and subscales estimates

Group	Baseline	3 months	6 months	9 months	12 months	Change baseline—12 months			*P* value
	Estimate	Estimate	Estimate	Estimate	Estimate	Estimate	Percent (%)	Cohen’s d	
*EDEQ total*									
CAU	1.8	2.2	2.4	2.5	2.6	+ 0.8	+ 47.5	0.66	0.007
LI	1.9	1.9	1.8	1.8	1.8	− 0.1	− 4.2	− 0.06	
LI without SMS	1.9	1.8	1.7	1.7	1.6	− 0.3	− 13.0	− 0.19	0.399
LI with SMS	1.9	2.0	2.0	2.0	2.0	+ 0.1	+ 5.35	0.08	
*EDEQ restraint*									
CAU	1.7	2.3	2.6	2.7	2.8	+ 1.1	+ 62.8	0.81	0.254
LI	1.6	1.9	2.0	2.1	2.1	0.6	+ 37.7	0.44	
LI without SMS	1.5	1.6	1.6	1.6	1.6	+ 0.1	+ 7.2	0.08	0.015
LI with SMS	1.6	2.3	2.6	2.8	2.9	+ 1.3	+ 82.8	1.0	
*EDEQ shape concern*									
CAU	2.3	2.7	2.9	3.0	3.0	+ 0.8	+ 35.1	0.45	0.016
LI	2.5	2.3	2.2	2.2	2.1	− 0.3	− 14.0	− 0.20	
LI without SMS	2.5	2.3	2.2	2.2	2.1	− 0.4	− 15.8	− 0.22	0.992
LI with SMS	2.5	2.3	2.2	2.1	2.1	− 0.4	− 15.7	− 0.22	
*EDEQ weight concern*									
CAU	2.3	2.8	3.0	3.1	3.2	+ 0.9	+ 41.8	0.57	0.007
LI	2.4	2.3	2.2	2.2	2.1	− 0.3	− 11.3	− 0.17	
LI without SMS	2.4	2.2	2.1	2.1	2.0	− 0.4	− 15.8	− 0.23	0.790
LI with SMS	2.4	2.3	2.2	2.2	2.2	− 0.2	− 9.5	− 0.14	
*EDEQ eating concern*									
CAU	0.9	1.1	1.2	1.3	1.4	+ 0.5	+ 56.1	0.43	0.116
LI	1.0	1.0	1.0	1.0	1.0	0.0	+ 2.6	0.02	
LI without SMS	0.9	0.9	0.9	0.9	0.9	0.0	+ 1.9	0.02	0.954
LI with SMS	1.1	1.1	1.1	1.1	1.1	0.0	+ 3.7	0.04	

*EDEQ* Eating Disorder Examination Questionnaire, *CAU* Care as Usual, *LI* lifestyle, *LI without SMS* lifestyle without short message service, *LI with SMS* lifestyle with short message service, Cohen’s D: 0.20 = small effect, 0.50 = medium effect and 0.80 = a large effect

### Changes in DEBQ scores

The DEBQ subscales emotions (Cohen’s d: − 0.23; P = 0.311), restraint (Cohen’s d: − 0.09; *P* = 0.761) and external eating (Cohen’s d: − 0.10; *P* = 0.675) did not change significantly in CAU compared to LI. Also, the two dimensions diffuse emotions (Cohen’s d: − 0.30; *P* = 0.181) and clearly defined emotions (Cohen’s d: − 0.18; *P* = 0.457) did not change significantly in CAU compared to LI. The same pattern was found if we compared LI without SMS to LI with SMS: emotions (Cohen’s d: − 0.18; *P* = 0.471), restraint (Cohen’s d: 0.26; *P* = 0.372) and external eating (Cohen’s d: 0.40; *P* = 0.142). The two dimensions for emotional eating, diffuse emotions (Cohen’s d: 0.04; *P* = 0.855) and clearly defined emotions (Cohen’s d: − 0.28; = 0.296) did not change significantly between LI with or without SMS, Table 3.

**Table 3:** DEBQ subscales estimates

Group	Baseline	3 months	6 months	9 months	12 months	Change baseline—12 months			*P* value
	Estimate	Estimate	Estimate	Estimate	Estimate	Estimate	Percent (%)	Cohen’s d	
DEBQ *emotions*									
CAU	3.2	3.3	3.4	3.4	3.5	− 0.2	− 8.3	− 0.20	0.311
LI	3.2	3.3	3.3	3.4	3.4	− 0.4	− 15.2	− 0.44	
LI without SMS	3.1	3.2	3.2	3.2	3.3	− 0.4	− 13.4	− 0.36	0.471
LI with SMS	3.2	3.4	3.5	3.6	3.6	− 0.5	− 19.6	− 0.53	
DEBQ *diffuse emotions*									
CAU	2.6	2.5	2.5	2.4	2.4	− 0.2	− 6.1	− 0.14	0.181
LI	3.1	2.8	2.7	2.6	2.6	− 0.5	− 15.9	− 0.45	
LI without SMS	3.0	2.7	2.6	2.5	2.5	− 0.5	− 17.0	− 0.44	0.855
LI with SMS	3.1	2.9	2.8	2.7	2.7	− 0.5	− 14.7	− 0.40	
DEBQ *clearly defined emotions*									
CAU	2.2	2.1	2.1	2.0	2.0	− 0.2	− 9.3	− 0.22	0.457
LI	2.6	2.4	2.3	2.3	2.2	− 0.4	− 14.4	− 0.39	
LI without SMS	2.6	2.4	2.4	2.3	2.3	− 0.3	− 11.1	− 0.28	0.296
LI with SMS	2.6	2.3	2.2	2.1	2.1	− 0.6	− 21.7	− 0.57	
DEBQ *restraint*									
CAU	3.2	3.3	3.4	3.4	3.5	+ 0.3	+ 9.8	0.35	0.761
LI	3.2	3.3	3.3	3.4	3.4	+ 0.2	+ 7.4	0.26	
LI without SMS	3.1	3.2	3.2	3.2	3.3	+ 0.1	+ 4.8	0.19	0.372
LI with SMS	3.2	3.4	3.5	3.6	3.6	+ 0.4	+ 10.9	0.45	
DEBQ *external*									
CAU	2.6	2.5	2.5	2.5	2.5	− 0.1	− 4.8	− 0.22	0.675
LI	2.9	2.8	2.7	2.7	2.7	− 0.2	− 6.3	− 0.32	
LI without SMS	2.9	2.8	2.7	2.7	2.6	− 0.3	− 9.5	− 0.49	0.142
LI with SMS	2.8	2.8	2.8	2.8	2.8	− 0.1	− 1.9	− 0.09	

*DEBQ* dutch eating behavior questionnaire, *CAU* care as usual, *LI* lifestyle, *LI without SMS* lifestyle without short message service, *LI with SMS* lifestyle with short message service, Cohen’s D: 0.20 = small effect, 0.50 = medium effect and 0.80 = a large effect

### Mediation of androgens, weight and depression

**Fig. 2:**
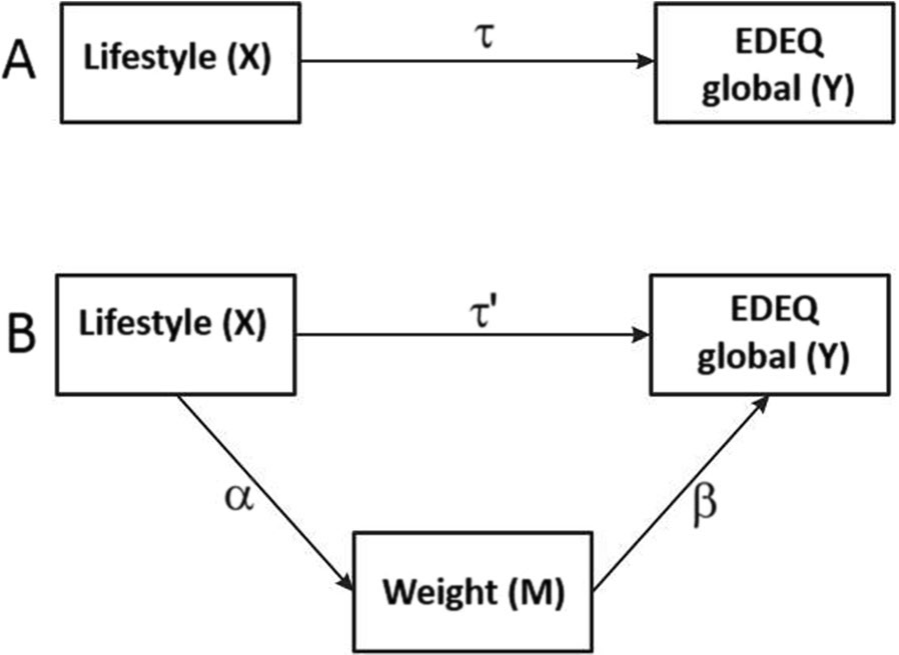
Mediation analysis

A mediating variable M is a variable that lies within the causal chain between an independent variable X and a dependent variable Y and represents the mechanism of change (Fig. 2). Panel A indicates a hypothetical causal relationship in which the lifestyle intervention (X) affects eating behavior measured with the EDEQ global score (Y). In Panel B, this relationship is hypothesized to be mediated: the lifestyle intervention (X) is hypothesized to reduce weight (M), which in turn would reduce EDEQ global scores (Y). In this way, the effect of the lifestyle intervention on eating behavior should primarily take place through paths a and b, rather than through path τ′. As a result, we found no mediation in the relationship between lifestyle and EDEQ global scores with weight as a mediator (*P* = 0.832). We also tested 9 other potential mediators (testosterone, androstenedione, dehydro-epiandrosterone (DHEA), insulin, HOMA-IR, cortisol, oligomenorrhea and depression. We found no mediation by these variables in the relationship between lifestyle and EDEQ global scores over time. These results indicate that metabolic features of PCOS, sex steroids, weight and psychological measures were not involved in the observed effects of LI on eating behavior over time.

## Discussion

To the best of our knowledge, we performed one of the largest RCT investigating weight loss during a three-component CBT LI intervention in women with PCOS. Several one- or two component interventions achieved short-term weight loss in women with PCOS [52] and did not examine eating behavior. We thus performed an analysis of the eating behavior data that was collected in the RCT. In accordance with our hypothesis, disordered eating behavior improved during a three-component LI program which combined nutritional advice, exercise and cognitive behavioral therapy while women in CAU developed more disordered eating behavior. We found a medium to large effect size (expressed in Cohen’s D) for the changes in eating behavior when we compared the effects of LI to CAU. This suggest that the lifestyle intervention is more effective than CAU to change eating behavior in women with PCOS. During our three-component LI intervention, women lost weight [53] while disordered eating behavior improved. Suggesting that the combination of the three-components are also effective for changes in eating behavior. During the study period, no differences in eating behavior were observed in the LI intervention with SMS compared to the LI without SMS. Suggesting that the additional SMS was not more effective for changes in eating behavior than the regular LI. We only found an exception for the EDEQ restraint subscale, whereas LI with additional SMS resulted in more dietary restraint. Based on previous studies, the relationship between dietary restraint and weight remains inconclusive. In some studies, dietary restraint was associated with a higher BMI [54] but also with a higher intake of healthy foods [55]. In line with our results, a study by Keränen and colleagues showed that restraint behavior increased more during a lifestyle intervention than in the control group. Especially in successful participants there was an increase in dietary restraint and a decrease in emotional and uncontrolled eating [56]. In a lifestyle intervention performed by Nurkkala and colleagues, cognitive restraint improved more in a lifestyle intervention than in the control group [57]. Some researchers suggested that a rigid and a flexible form of dietary restraint exist [58]. The rigid form was associated with a more disordered eating pattern and binge eating. The flexible form was characterized by a less disordered eating pattern and more weight loss success. In our study, dietary restraint increased both in CAU and in LI with SMS while the latter resulted in the highest amount of weight loss [38]. Therefore, it could be speculated that the increase in dietary restraint was positive for participants in the LI group but not for the CAU group. Suggesting that the strict and flexible form of restraint eating might have played a role in this group of women. Unfortunately, the two forms of restraint eating can only be examined with the Eating Inventory and not by the EDEQ or DEBQ questionnaires that we have used in this study. Therefore, we cannot conclude if the flexible form of restraint eating improved in the LI and worsened in CAU to explain the differences we found.

Within the general population, risk factors for disordered eating were associated with psychosocial, demographic, environmental and genetic factors [59–61]. It is unclear why so many women with PCOS have disordered eating behaviors. The current literature suggests that distress, low self-esteem [62] and depression [32] were associated with disordered eating in women with PCOS. This is in line with the general population where higher depression scores were related to eating disorders [63]. Therefore, we tested different mediators in the relationship between lifestyle treatment and changes in disordered eating behavior. Surprisingly, we found no significant mediation by weight or depression scores that could explain the changes in eating behavior during the lifestyle treatment. We also tested other potential mediators since a relationship between high levels of androgens and binge eating was found in the general population [64]. In women with PCOS, a connection between high androgen levels, polycystic ovaries and behavioral deficits such as impulsivity was suggested, which could make women with PCOS more vulnerable for bulimia nervosa [65]. Others also suggested that the irregular menstrual cycle (oligomenorrhea) [66, 67] or high levels of insulin [30] may lead to increased hunger and psychological distress, which could result in more binge eating. However, we found no mediation by weight, depression, androgens, insulin, HOMA-IR, cortisol or oligomenorrhea. This could suggest that the lifestyle intervention itself and not depression, weight or androgens were involved in the changes in eating behavior that were observed during the lifestyle intervention.

A limitation of the present trial is that we observed high drop-outs rates comparable to other obesity treatments in the general population [68, 69]. In lifestyle programs designed for women with PCOS drop-out rates of around 25% were reported. It is still unclear if patient or intervention related factors are related to drop-out [70]. Drop-outs could effected the results of many lifestyle interventions because outcomes were most of the times based on complete cases analyses. Complete cases analyses can overestimate weight loss because study completers achieve more weight loss than drop-outs [71]. To prevent these overestimations, we have chosen a statistical method that included all available data even if participants dropped out during the study period. Despite an overall drop-out rate of 63.4%, the mixed multilevel model was based on a high number of measurements (394 in total) belonging to 183 participants.

Future research should examine whether women with PCOS and different types of eating behavior benefit from surgical or nonsurgical weight loss interventions. Based on the mechanisms and etiology of the different eating behaviors, a different treatment seems required for each behavior to achieve weight loss [22]. At the moment, we are performing a new RCT to test the effects of gastric bypass surgery versus the current three-component lifestyle intervention in women with PCOS. Especially to examine which treatment works best for this large and diverse group of women.

## Conclusions

Treatment by a three-component lifestyle program that combined nutritional advice, exercise and CBT resulted in a medium to large effect size and significant improvements in disordered eating behavior compared to CAU. Neither weight loss, depression, testosterone, androstenedione, DHEA, insulin, HOMA-IR nor cortisol did mediate this effect. A multidisciplinary lifestyle treatment is effective to improve disordered eating behavior in women with PCOS.

## Data Availability

The datasets used and/or analysed during the current study are available from the corresponding author on reasonable request.
